# Motor learning of novel dynamics is not represented in a single global coordinate system: evaluation of mixed coordinate representations and local learning

**DOI:** 10.1152/jn.00493.2013

**Published:** 2013-12-18

**Authors:** Max Berniker, David W. Franklin, J. Randall Flanagan, Daniel M. Wolpert, Konrad Kording

**Affiliations:** ^1^Rehabilitation Institute of Chicago, Northwestern University, Chicago, Illinois;; ^2^Computational and Biological Learning Lab, Department of Engineering, University of Cambridge, Cambridge, United Kingdom; and; ^3^Department of Psychology and Centre for Neuroscience Studies, Queen's University, Kingston, Ontario, Canada

**Keywords:** motor control, motor adaptation, intralimb generalization, coordinate frames, internal models

## Abstract

Successful motor performance requires the ability to adapt motor commands to task dynamics. A central question in movement neuroscience is how these dynamics are represented. Although it is widely assumed that dynamics (e.g., force fields) are represented in intrinsic, joint-based coordinates (Shadmehr R, Mussa-Ivaldi FA. *J Neurosci* 14: 3208–3224, 1994), recent evidence has questioned this proposal. Here we reexamine the representation of dynamics in two experiments. By testing generalization following changes in shoulder, elbow, or wrist configurations, the first experiment tested for extrinsic, intrinsic, or object-centered representations. No single coordinate frame accounted for the pattern of generalization. Rather, generalization patterns were better accounted for by a mixture of representations or by models that assumed local learning and graded, decaying generalization. A second experiment, in which we replicated the design of an influential study that had suggested encoding in intrinsic coordinates (Shadmehr and Mussa-Ivaldi 1994), yielded similar results. That is, we could not find evidence that dynamics are represented in a single coordinate system. Taken together, our experiments suggest that internal models do not employ a single coordinate system when generalizing and may well be represented as a mixture of coordinate systems, as a single system with local learning, or both.

how the central nervous system represents information about the body and the environment for the purposes of skilled action is one of the central questions in sensorimotor neuroscience (Franklin and Wolpert 2011; Scott 2012). In a now classic study, Shadmehr and Mussa-Ivaldi (1994) investigated how subjects represent novel dynamics while making reaching movements in a robot-rendered force field. By proposing two categorically distinct hypotheses of extrinsic (i.e., Cartesian based) and intrinsic (i.e., joint based) coordinate frames, the authors made clear predictions for how subjects would generalize when the posture of their limb changed. After learning to reach in the force field in one region of the workspace, subjects were then tested for their ability to generalize in a second region. The results led the authors to conclude that the subjects represented the force field in intrinsic coordinates.

Numerous subsequent studies have examined adaptation and generalization in this framework, designing experiments and interpreting results in the context of extrinsic versus intrinsic coordinate frames. Unfortunately, the results found through this effort have not offered a unified view for internal representations. Depending on the kind of motor behavior subjects adapt to (e.g., force field, inertial perturbation, visuomotor rotation) and the type of generalization examined (e.g., interlimb or intralimb), evidence for intrinsic variables (Malfait et al. 2002, 2005; Shadmehr and Moussavi 2000) and extrinsic variables (Burgess et al. 2007; Criscimagna-Hemminger et al. 2003; Krakauer et al. 2000) has been obtained. Indeed, recent studies of visuomotor adaptation have suggested that learning may be represented as a mixture of both intrinsic and extrinsic coordinate systems (Brayanov et al. 2012). The collective results suggest that internal models may not rely on an easily categorical representation.

Although Shadmehr and Mussa-Ivaldi (1994) found evidence that dynamics of the arm are represented in intrinsic coordinates, this study only tested generalization across changes in shoulder angle. If dynamics are represented in joint-based coordinates, then appropriate generalization should be seen with changes in other joints (e.g., the elbow and wrist). Furthermore, the original results are consistent with encoding of dynamics in object-centered coordinates. Subjects might interpret the dynamics as belonging to a grasped object, where the dynamics will rotate with the orientation of the hand. This would give the appearance of joint-based coordinates if only the shoulder was rotated. Finally, despite many similar studies having been performed, the original study has never been reproduced. We therefore reexamined the hypothesis of extrinsic versus intrinsic representations.

In a first experiment, we examined transfer of force field learning to novel arm configurations involving changes in the shoulder, elbow, and wrist angles. This enabled us to test whether subjects generalize so as to produce forces that are identical in joint space, in Cartesian space, or in a coordinate frame that rotates with the hand's orientation, respectively. Subjects adapted to a force field and were then probed for generalization, making reaches in force channels. We found that generalization was not consistent with any of the three individual coordinate frames. However, the pattern of generalization could be well accounted for by a model that used a linear combination of the three as well as when using several reduced models that included spatial decay, i.e., local learning. To examine whether details of the experimental paradigm could account for our failure to support the original study, our second experiment was a reproduction of the original study. Here again, we found that although subjects learned to predict the force field during adaptation, their performance when generalizing failed to support either an intrinsic or an extrinsic encoding of novel dynamics. The results from the two experiments argue against the idea that dynamics are represented in any single coordinate frame.

## MATERIALS AND METHODS

*Experiment 1*, which was conceived by D. W. Franklin, J. R. Flanagan, and D. M. Wolpert and run in Cambridge, tested generalization in extrinsic, intrinsic, and object-centered coordinates by probing generalization across a wide range of joint configurations. *Experiment 2*, which was run in Chicago by M. Berniker and K. Kording, reproduced the study by Shadmehr and Mussa-Ivaldi (1994) along with two variations on the original protocol. Although the two experiments were run independently without knowledge of the other experiment, they are complementary studies that examine the same issue: which coordinate frames are used during motor adaptation and generalization.

### Experiment 1

Nine naive right-handed subjects (7 men, 2 women) were recruited to take part in the experiment (mean and SD of age: 22.8 ± 2.8 yr). All subjects were right-handed according to the Edinburgh handedness inventory (Oldfield 1971) and had no reported neurological disorders. Subjects gave informed consent, and the institutional ethics committee approved the experiments. At the beginning of the experiment, each subject's limb lengths and shoulder position were measured for use in the experimental program.

#### Experimental apparatus.

**Fig. 1.**
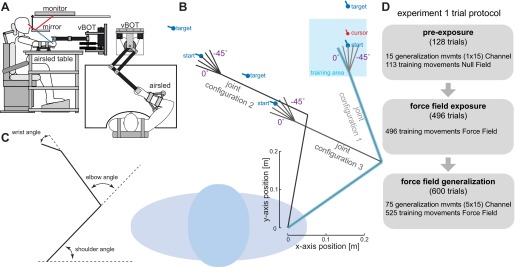
Experimental protocol for *experiment 1*. *A*: experimental setup in which subjects grasp the handle of a robotic manipulandum. Also shown is the air sled table that subjects rested their arm on and the monitor-mirror system that provided visual feedback. *B*: training and generalization postures and movements. The posture is composed of the joint configuration (shoulder and elbow angles) and the hand orientation (which is varied by changing the wrist angle). The training posture (blue arm) defined the 15 × 15 cm training workspace (blue shaded square). All training movements were performed with the hand oriented in the workspace as shown. The transfer of learning to other locations was examined at 15 postures (gray arms). In *joint configuration 1*, the hand could be oriented at 5 angles and a 10-cm generalization movement was performed directly ahead at each posture. The start, target, and cursor for the generalization movement at the middle hand orientation are shown. Similarly, 5 other hand orientations (each associated with a single generalization movement direction) were used for *joint configuration 2* and *joint configuration 3*. Again, the movement for the middle hand orientation is illustrated with the start and target locations. All generalization movements were performed in a simulated mechanical channel in which the force produced by the subjects could be measured against the channel wall. The orientation of the hand in Cartesian space was always consistent for both start and end targets for any given movement. *C*: illustration of the 3 joint angles, which are calculated relative to the previous segment orientation. *D*: outline of the 3 stages of *experiment 1* detailing the number and type of trials that occur in each stage.

The physical environment was generated with the vBOT robotic manipulandum (Howard et al. 2009) (Fig. 1*A*). Position and force data were sampled at 1 kHz. End-point forces at the handle were measured with an ATI Nano 25 six-axis force-torque transducer (ATI Industrial Automation). The position of the vBOT handle was calculated from optical encoders on the motors. Visual feedback was provided with a computer monitor mounted above the vBOT and projected veridically to the subject via a mirror. This virtual reality system covers the manipulandum and the arm and hand of the subject, preventing direct visual feedback of the hand location. The position and orientation of the hand, forearm, and upper arm in Cartesian space were measured with an Optotrak (Northern Digital) and sampled at 100 Hz. This was performed by fixing a rigid block onto the flat portion of the back of the subject's right hand as it grasped the handle of the vBOT. This rigid block contained six Optotrak markers (3 on one side and 3 on a side oriented 90° to the first side). Throughout the experiments, at least three of the markers were visible at all times to the Optotrak system. Similarly, both the position and orientation of the forearm and upper arm were recorded with three markers fixed on a rigid block to each limb segment. The relative shoulder, elbow, and wrist angles were calculated in real time based on the measured limb lengths and shoulder position, while the hand orientation was used for feedback and control in the experiment. Visual feedback of targets (1.6-cm disks) and the hand (1.2-cm cursor) was updated at 60 Hz.

#### Experimental procedure.

After a brief session designed to familiarize subjects with the equipment, they performed three stages of the experiment. The first stage was a preexposure stage in which we characterized baseline performance prior to any force field learning. This was followed by an exposure stage in which all movements were performed in a force field but limited within a training workspace. Finally, in the generalization phase, we continued exposure to the force field in the training workspace while assessing generalization of learning at different arm configurations using channel trials.

Subjects performed 10-cm reaching movements with their right arm while grasping a robotic manipulandum. The reaching movements were confined to the horizontal plane ∼10 cm below the subjects' shoulder level. The forearm was supported against gravity with an air sled (Fig. 1*A*). The hand cursor was displayed as a circular disk (1.2-cm cursor) with an oriented bar that displayed the orientation of the hand. Similarly, the start posture and final target posture were specified by circles (1.6-cm disks) with bars indicating orientation that was the same for both start and target (Fig. 1*B*). Prior to movement, the hand was required to be stationary within the start target (positional tolerance 0.5 cm) and the orientation bars coaligned (hand orientation tolerance 5°). Once the criteria had been achieved for 800 ms, a tone indicated that the subject could initiate the trial by moving to the final target posture. Movements were required to be made to the target (final positional tolerance 0.8 cm and hand orientation tolerance 12.5°) within 400 ± 75 ms, and if this was achieved feedback was given (“good” or “great”) and a (motivational) counter increased by 1. Otherwise, appropriate feedback was given (“too slow,” “too fast”).

Exposure to the force field took place within a 15 cm × 15 cm training area centered at the training posture (Fig. 1*B*). The training posture was defined with a shoulder joint angle of 35°, an elbow joint angle of 75°, and the wrist joint angle set at an appropriate posture for each subject (mean ± SD: 12.3 ± 11.6°) (Fig. 1*B*). This posture corresponded to the training posture used in Shadmehr and Mussa-Ivaldi (1994). The wrist joint angle for this training posture was chosen separately for each subject to ensure that all 15 test movements (described below) could be performed comfortably without approaching joint limits. As in Shadmehr and Mussa-Ivaldi (1994), movements were in eight equally spaced directions (with straight ahead at 0°), with each subsequent movement starting from the end of the previous movement and the direction chosen randomly so that the movements were always constrained within the training area. It is worth noting that, unlike the great majority of force field studies, subjects did not adapt to a sequence of eight center-out reaches. Rather, the sequence of targets formed a pseudorandom walk within the training workspace. As such, although the same reaching directions were repeated many times, the reach locations themselves were rarely repeated.

During exposure movements we applied a velocity-dependent force field (Shadmehr and Mussa-Ivaldi 1994), where the forces on the handle depended on the Cartesian velocity (*x˙*) of the end point of the limb:

(1) $$F=B\overset{˙}{x},$$

where ***F*** is the force vector, **x** = [*x*,*y*]^*T*^ is vector position of the subject's hand, and ***B*** is a matrix defining the mapping from velocities to forces. As in the original study (Shadmehr and Mussa-Ivaldi 1994) we defined ***B*** to be

$$B=\left[\begin{array}{cc} -10.1 & -11.2\\ -11.2 & 11.1 \end{array}\right]\text{Ns/m}$$

Note that, unlike a curl field, this matrix defines a force field that has unique axes. As such, movements in different directions give rise to different perturbing forces. This allowed us to make distinct predictions for different directions and joint configurations during generalization.

In both the preexposure and generalization stages of the experiment we used channel trials to assess the force compensation during movement at different configurations of the shoulder, elbow, and wrist. The shoulder and elbow angles were chosen to be [35°, 75°] (*joint configuration 1*), [35°, 120°] (*joint configuration 2*), or [80°, 75°] (*joint configuration 3*); that is, with either a 45° elbow or shoulder flexion, respectively (see Fig. 1*B*). For each of these three joint configurations, the hand was oriented at one of five angles (each separated by 11.25°) relative to the wrist angle at the training posture [0°, −11.25°, −22.5°, −33.75°, −45.0°]. This gives a total of 15 test limb postures and corresponding test movements (Fig. 1*B*). By exploring a larger range of limb postures, as well as wrist orientations, we could explore the coordinates used during generalization. Prior to the start of the experiment, we measured the lengths of each subject's hand, forearm, and upper arm. By combining this information with the current hand position and hand orientation, the location of the subject's wrist was obtained in real time. All target locations for the 15 test movements were generated with these measurements to ensure that the shoulder and elbow joints were matched across the five hand orientations for each of the configurations.

All generalization movements were performed in a mechanical channel that constrained movement along a straight line to the target. The virtual mechanical channel was simulated as a one-dimensional spring and damper (stiffness 5,000 N/m and damping 2 N·m^−1^·s^−1^) orthogonal to the direction of movement (Milner and Franklin 2005; Scheidt et al. 2000). Visual feedback of the hand position and orientation was available throughout this test movement. Many studies have demonstrated that the force produced against such force channels on random trials during learning grows steadily as subjects learn to compensate for the dynamics, approaching 80% of that required for complete adaptation (Howard et al. 2012, 2013; Smith et al. 2006). Prior to each movement the hand was moved passively by the robot [using a minimum jerk motion (Flash and Hogan 1985)] to the start target of the test movement, and afterwards the hand was again moved passively back to the start target of the subsequent movement within the training area. Previous studies have demonstrated that when subjects repeatedly move in a mechanical channel there is very little trial-by-trial decay in force production (Scheidt et al. 2000). However, to ensure that learning of the force field in the training position was maintained, generalization movements were always followed by an exposure movement in the training workspace.

For each configuration, we designed the movements so that for the five hand orientations the initial position and translation of the wrist joint were identical (see Fig. 1*B*), ensuring that for these movements the shoulder and elbow joint rotational trajectories were similar. To control the hand orientation in Cartesian space we required subjects to adopt correct wrist orientation prior to the beginning and at the end of the trial.

The preexposure stage consisted of 128 trials in total, with 113 movements in the null field and 15 generalization movements (1 repetition of each test movement) with a mechanical channel. The exposure stage consisted of 496 force-field movements in the training workspace. In the generalization stage, subjects performed an additional 600 trials. This was a pseudorandomly ordered mixture of 525 exposure movements in the training workspace and 75 generalization movements in the mechanical channel (5 repetitions of each of the 15 test movements).

#### Data analysis and models.

The data analysis was performed in MATLAB 2012a (The MathWorks). Trials were aligned to movement onset (velocity first exceeds 0.5 cm/s) for analysis. The effects and learning of the force field within the exposure movements were examined using the maximum perpendicular error (MPE) from the straight line between the start and target locations. The peak speed in the generalization movement in the channel was calculated as the maximum velocity during the movement. To examine the generalization of the learned forces across the tested limb postures, the end-point force on the trials in the mechanical channel was measured and the mean of the force was calculated between 50 and 450 ms. For comparison against model predictions, for each generalization movement the channel trial force in the preexposure stage was subtracted from the channel trial force in the generalization stage. This allowed us to assess what subjects learned relative to the preexposure stage. Limb posture was defined as the joint configuration (the particular shoulder and elbow angles) and the hand orientation (the orientation of the hand in external space). Statistical tests were performed with the general linear model in SPSS (Statistics 21, IBM) with subjects as a random factor. Statistical significance was considered at the *P* < 0.05 level.

We considered two model classes to explain the pattern of generalization. The first class examines global generalization models based on three different coordinate systems: joint, Cartesian, and object, as well as mixtures of these coordinate systems. The second class examines local learning models in which the pattern of generalization is determined by the same three coordinate systems or their mixtures but with spatial decay away from the training workspace.

#### Model predictions—global generalization models.

We considered three possible representations of dynamics that make different predictions with regard to generalization in our experiment. In the experiment, subjects learn to compensate for a force field of the form ***F*** = ***B***x˙. All exposure occurs in one training location, defined by a training limb posture. When subjects move to a new posture we can determine the matrix, ***B***_gen_, that would be expected based on generalization in the three different coordinate frames.

**Fig. 2.**
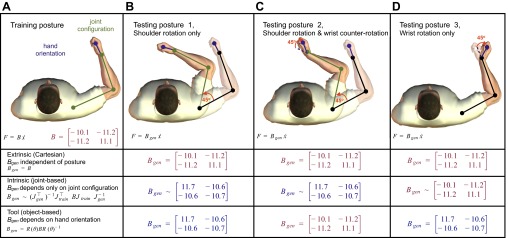
Schematic of the effect of the representation of dynamics on the pattern of generalization to novel postures. *A*: subjects learn to compensate for novel dynamics (external force field *B*) in a training posture defined by the joint configuration (shoulder and elbow angles) and the hand orientation. The generalization can then be tested at different limb postures in order to probe the coordinate frame in which the representation of the dynamics has been learned. Each of these 3 possibilities, extrinsic, intrinsic, and tool based, make different predictions depending on the posture. *B*: in *testing posture 1*, the arm has been rotated around the shoulder by 45°. While the predicted generalization of the force field (*B*_gen_) for an extrinsic (Cartesian based) representation is unchanged, the predictions from both intrinsic and object-based representations are different (but identical to each other). This limb posture is equivalent to that examined in Shadmehr and Mussa-Ivaldi (1994). Note that it is not possible to distinguish object-based from intrinsic representations using only this posture. *C*: in *testing posture 2*, the wrist angle has been counterrotated by 45° (relative to *testing posture 1*) such that the hand orientation is identical to the training posture. Now the predictions based on extrinsic and object-based representations are identical but different from those based on an intrinsic representation. Note that θ in the rotation matrix corresponds to hand orientation in external space and not to wrist angle. *D*: finally, when only the hand orientation is changed by 45° (*testing posture 3*) from the training posture, the extrinsic and intrinsic predictions are identical and only the prediction from the object-based representation is different. For the intrinsic (joint based) predictions, the ∼ indicates that we model the arm here as a 2-joint system for illustrative purposes (although all modeling and analysis is performed with a 3-joint model; see materials and methods for details).

With a Cartesian representation, forces and the corresponding matrix are invariant with respect to limb posture (e.g., Fig. 2) and *B*_gen_ = *B* (red refers to matrices that are the same as the training posture matrix). If subjects generalize the force field in Cartesian coordinates, they would produce the same compensation at each of the three joint configurations. Note that the movement directions for *joint configurations 2* and *3* are the same and lead to identical predictions. Therefore, for any given movement direction, in each of the three joint configurations the predicted forces are independent of shoulder, elbow, or wrist orientation.

With a joint-based representation, forces should change with the limb's posture (Fig. 2). In contrast to the original study, we required subjects to maintain a constant wrist orientation during each movement. We considered three possible ways in which the participants could encode the experienced torques in joint-based coordinates. First, as in the original study, we considered the wrist as a fixed joint such that for joint-based learning subjects learn a mapping of shoulder and elbow velocities to shoulder and elbow torques. Second, we considered a modified model in which the forearm and hand act as a single (virtual) segment from elbow to hand. This was done because in generalization trials the orientation of the wrist slightly influences the mapping between shoulder and elbow torques and the corresponding forces at the handle (i.e., the Jacobian). Both these two models use a 2 × 2 Jacobian relating hand velocity to joint velocities. Third, we considered a joint-based representation in which subjects interpret the three joint torques as depending on the shoulder, elbow, and wrist angular velocities and that they generalize in this three-dimensional joint space. That is, we use a 2 × 3 Jacobian relating hand velocity to the shoulder, elbow, and wrist joint angular velocities. Examination of these three models showed they had very similar predictions and the use of any led to the same conclusions when analyzing the data; therefore, for simplicity we only describe the last model (and use it to fit the data), in which subjects represent the field as a function of all three joint angular velocities.

Although the two-segment models allow us to calculate an equivalent *B*_gen_ (which we display in Figs. 1 and 5), because of the noninvertibility of the three-joint Jacobian we cannot calculate an equivalent *B*_gen_ for this model. However, we can predict the forces that we expect on the generalization trials based on this three-joint intrinsic coordinate representation, which allows us to test the model. Let *J*_train_ be the limb's Jacobian at the training configuration, ***F*** the force vector in Cartesian coordinates, and τ the torque vector in joint (shoulder, elbow, and wrist) coordinates, *q˙* = [*q˙*_s_,*q˙*_e_,*q˙*_w_]^*T*^. Given that τ = *J*^*T*^*F* and *x˙* = *Jq˙*, forces can be converted into an equivalent joint-based representation of torques as follows:

(2) $$\tau =J_{\text{train}}^{T}F=J_{\text{train}\;}^{T}B\overset{˙}{x}=J_{\text{train}}^{T}\;BJ_{\text{train}}\overset{˙}{q}=W\overset{˙}{q}$$

where ***W*** is the analog of the matrix ***B*** mapped to the joint-based reference frame. Assuming this is the representation subjects learn, we can compute the expected forces for test movements. Given the basic properties of the transformation from end-point forces to joint torques [τ = *J*^*T*^*F* → *F* = (*JJ*^*T*^)^−1^*J*τ], this gives

(3) $$F={\left(J_{\text{test}}J_{\text{test}}^{T}\right)}^{-1}J_{\text{test}}\tau ={\left(J_{\text{test}}J_{\text{test}}^{T}\right)}^{-1}J_{\text{test}}W\overset{˙}{q}\;={\left(J_{\text{test}}J_{\text{test}}^{T}\right)}^{-1}J_{\text{test}}J_{\text{train}\;}^{T}BJ_{\text{train}}J_{\text{test}}^{-\text{1}}\overset{˙}{q}$$

As our paradigm required the subjects to maintain a constant hand orientation during the movement, the angular velocity of the wrist is simply equal to the negative of the sum of the shoulder, elbow, and wrist velocities (Fig. 1*C*). This allows us to predict the time series of the expected force for any generalization movement by applying the appropriate varying Jacobian of the arm (with link lengths set for the average arm of 30, 25, and 7 cm for the upper, lower, and hand segments), taking into account the variation in shoulder, elbow, and wrist angle (Fig. 2).

Finally, with object-based coordinates, forces should rotate with the hand's orientation. Although the original study by Shadmehr and Mussa-Ivaldi (1994) concluded that generalization was in intrinsic coordinates, there is another possibility consistent with their findings, namely, that subjects interpret the dynamics as belonging to a grasped object and therefore expect the dynamics to rotate with the orientation of the hand. If subjects generalize in object-based coordinates, they will expect *B*_gen_ to be different from *B* if the hand's orientation in space has rotated by some angle θ (e.g., Fig. 2*B*). The precise form of *B*_gen_ depends on the original *B* and the rotation matrix *R*(θ) that defines the rotation of the hand between the training and test postures (Fig. 2*D*). That is, for a given orientation of the hand we first determine for a given hand velocity the force that would have been produced on the handle had the hand been in the training posture, which is given by *BR*(θ)^−1^*x˙*. The force expected at the new posture is now calculated by rotating these forces to the new hand orientation:

(4) $$F=R\left(\theta \right)BR{\left(\theta \right)}^{-1}\overset{˙}{x}=B_{\text{gen}}\overset{˙}{x}$$

This third coordinate frame allowed us to make predictions for how subjects would generalize when the hand orientation changed, independently of limb orientation, thereby further probing an object-based representation. Note that the angle θ denotes the hand orientation in external space and not the wrist joint angle.

Each of these three models predicts the forces we should observe for each of the generalization configurations (i.e., movements). We examined whether these predictions, as well as different mixtures of the predictions (i.e., mixed coordinate system representations) could account for the pattern of generalization seen in our subjects. That is, we considered a model in which generalization is as a mixture of the three coordinate systems

$$F=k_{\text{j}}J+k_{\text{c}}C+k_{\text{o}}O$$

where *F* is the force predicted by the model on channel trials, *J*, *C*, and *O* are the predictions of the single coordinate system models, and the *k* values are the mixture coefficients. We require the mixing coefficients to be positive (a negative coordinate system has little meaning) and sum to 1 (thereby guaranteeing that the models fully account for learning in the training posture). We fit all one-, two-, and three-coordinate system models (which had 0, 1, and 2 degrees of freedom, respectively) by constraining the appropriate *k* values to be zero in the model.

We performed two types of analysis. First, we fit the entire data set of generalization for our nine subjects using each of the different models. That is, we fit the generalization data with different linear combinations of the predictions of the three coordinate systems. For each of the subjects and for each of the three coordinate system predictions, we first scaled the forces so that at the training posture the force magnitude was unity. Therefore, the measures at the generalization postures are relative to what had been learned at the training posture. To compare the models we used the Bayesian information criterion (BIC):

BIC=n⋅ln(MSE)+k ln(n)

where MSE is the mean squared error of the model fit, *k* is the number of degrees of the model, and *n* is the total number of data points fit. The BIC allows models with different numbers of parameters to be compared—the one with a lower BIC is preferable. The difference in the BIC scaled by 0.5 approximates the log of the Bayes factor, the likelihood that one model is better than another (Kass and Raftery 1995). A Bayes factor larger than 10 indicates strong evidence in favor of a model, and a value larger than 100 is considered decisive (Jeffreys 1998).

Second, to examine individual subjects we used leave-one-out cross-validation. That is, for each subject we fit each model to the average of all the other subjects' data and used this fit to predict performance on the left-out subject. By using such “out of sample” cross-validation, we are able to examine the performance of each model without the need to correct for the different degrees of freedom of the models.

#### Model predictions—decaying generalization models.

We also considered decaying generalization models in which the pattern of generalization is determined by a single coordinate system (Cartesian, joint, or object-based coordinates) but the generalization shows decay in magnitude as a function of positional deviation from the training posture expressed in the metric of the same coordinate system. That is, for the Cartesian, joint, and object-based models, we examined distance metrics that were specified as Cartesian distance of the hand from the training hand location, three-dimensional Euclidean joint distance, and object orientation distance (the difference between the hand orientation in the training posture and in the test posture), respectively. In each case, the decay function is modeled in a Gaussian manner as a function of a distance metric. The equation for the three-component mixture model (J+C+O) with decay is given by

$$F=k_{\text{j}}Je^{-d_{\text{j}}\left(\Delta \theta_{\text{s}}^{2}+\Delta \theta_{\text{e}}^{2}+\Delta \theta_{\text{w}}^{2}\right)}+k_{\text{c}}Ce^{-d_{\text{c}}\left(\Delta x^{2}+\Delta y^{2}\right)}+k_{\text{o}}Oe^{-d_{\text{o}}\Delta \theta_{\text{hand}}^{2}}$$

where *F* is the force predicted by the models, *J*, *C*, and *O* are the predictions of the single-coordinate system models, the *k* values are the mixture parameters, which are constrained to be positive and sum to 1, and the *d* values are the decay parameters associated with distance from the training configuration in the three different coordinate systems. The decay depends on the difference from the training posture (Δ) measured in the same coordinate system as the generalization (change in shoulder, elbow, and wrist angle for joint based, change in Cartesian distance for Cartesian based, and change in hand orientation for object centered). We fit all one-, two-, and three-coordinate system decay models (which had 1, 3, and 5 degrees of freedom, respectively) by setting the appropriate *k* values to zero. Each of these models has a single parameter for each coordinate system determining the length scale of its decay. Again, we performed both a BIC and a cross-validation analysis.

### Experiment 2

In total, 30 right-handed subjects (29.2 ± 7.0 yr of age; 14 men, 16 women) took part in this experiment. All experimental protocols were approved by the Northwestern University Institutional Review Board and were in accordance with Northwestern University's policy statement on the use of humans in experiments. All participants were naive to the goals of the experiment, signed consent forms prior to participating, and were paid to participate.

#### Experimental procedure.

**Fig. 3.**
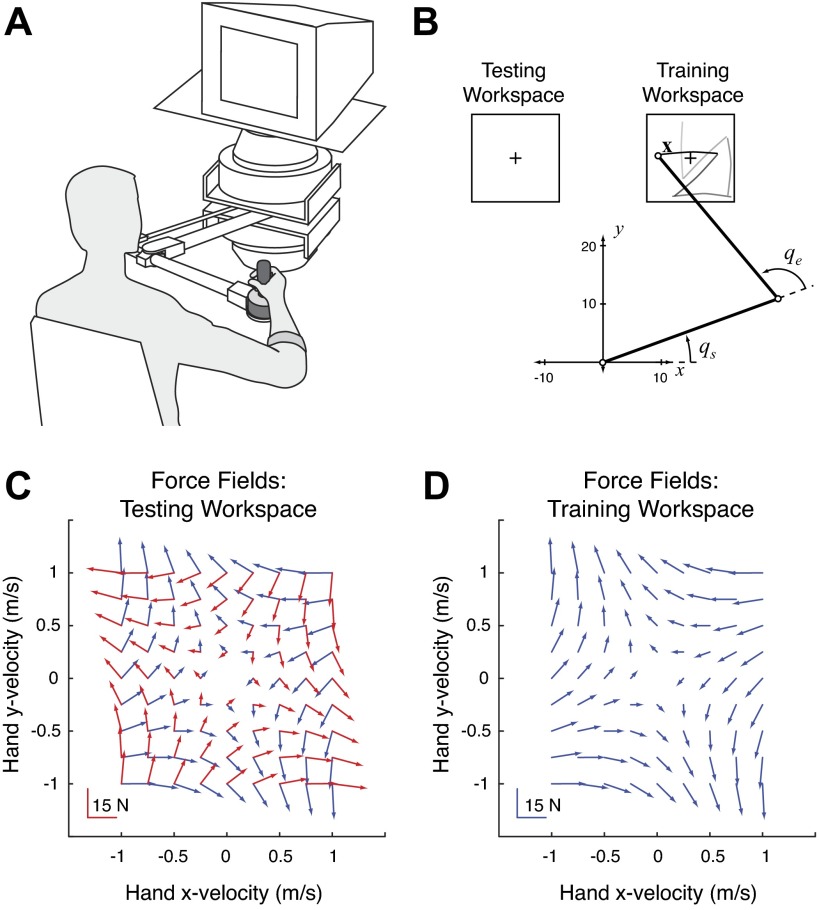
Experimental protocol for *experiment 2*. *A*: subjects gripped the handle of a robotic manipulandum with their arm in a sling (not shown) such that movements were in a plane approximately aligned with their shoulder. *B*: all reaching movements were made in the training and testing workspaces. These were located at a fixed distance from each subject's right shoulder (the origin). By measuring the location of their hand (the robot handle), their shoulder and elbow angles could be computed. All subjects adapted to a force field in the training workspace and were then probed for generalization in the testing workspace. *C* and *D*: the intrinsically and extrinsically defined force fields (red and blue vectors, respectively) were identical in the training workspace but nearly orthogonal in the testing workspace.

All subjects sat in a height-adjustable chair with their elbow in a suspended sling to reduce fatigue and ensure that their limb was approximately in a horizontal plane aligned with their shoulder (Fig. 3*A*). Subjects made reaches with their arm in two different configurations, defining a training workspace and a testing workspace (Fig. 3*B*) while grasping a robotic manipulandum. Each workspace was a 15-cm square within which all reaching targets were confined. To control for the anisotropy of the robot dynamics, the chair the subject sat in was moved relative to the robot to achieve these postures. Furthermore, these workspaces were uniquely defined for each subject based on his/her respective limb lengths (which were used to define subject-specific Jacobians, see below). In the training workspace, a subject's shoulder was ∼15 cm to the left and 40 cm in front of the center of the robot. In the testing workspace, the subject's shoulder was translated ∼30 cm to the left.

Subjects were instructed to make point-to-point reaching movements of a prescribed duration. Visual feedback was provided on a computer monitor, calibrated to display accurate displacements, sitting above the robot and in front of the subjects (Fig. 3*A*). A cursor displayed on the screen (a white circle 4 mm in diameter) depicted the location of their hand (i.e., handle of the manipulandum). Each trial began with the display of a new target (yellow circle 10 mm in diameter) randomly drawn from one of eight directions (0°, 45°, 90°, . . . 315°) and at a distance of 10 cm. If subjects came to a halt within the target, within 700 ± 100 ms of the trial's onset, then the target turned green, a tone was emitted, and their running score was advanced by 1. If they made the reach too slowly the target turned blue, and if too fast, red. After a brief pause, the target was extinguished and a new randomly drawn target was displayed. As in *experiment 1*, the sequence of targets formed a pseudorandom walk within the workspace.

During the course of the experiment, the robot would render different force fields applied to the subject's hand. As in the original study (Shadmehr and Mussa-Ivaldi 1994), and *experiment 1*, an extrinsic force field was defined in terms of the Cartesian velocity of the subject's hand using the matrix ***B*** (defined above). As described above, this force field is translation invariant with respect to the hand's coordinates and produces equivalent forces in the training and testing workspaces (Fig. 3, *C* and *D*). This force field was used to examine whether or not subjects would generalize from the training workspace to the testing workspace in extrinsic coordinates.

To test for the alternative, intrinsic hypothesis, an intrinsic force field was defined in terms of the subject's shoulder and elbow angular velocities: τ = ***W****q˙* (*Eq. 2*), where τ is the torque vector acting on the subject's shoulder and elbow joints, ***q*** = [*q*_s_, *q*_e_]^*T*^ is the limb's angular orientation, and ***W*** is a matrix relating joint velocities to torques. For similar reasons as outlined above, *Eq. 2* defines a force field that is translation invariant with respect to the limb's orientation. However, two reaches with identical hand displacements in the training and testing workspaces will produce different angular velocities and torques (Fig. 3, *C* and *D*). Thus this field would be ideal for testing whether or not subjects generalize in intrinsic coordinates.

Assuming subjects made reaches with minimal torso movement, there is a unique mapping from end-point forces to joint torques and from end-point velocity to joint velocity. Just as in *experiment 1*, with the location of a subject's hand and shoulder known, as well as their limb segment lengths, the force field that would accurately render *Eq. 2* is given by *F* = (*J*_test_^*T*^)^−1^*Wq˙* (*Eq. 3*), where *W* = *J*_train_^*T*^ *BJ*_train_ and *J*_test_ and *J*_train_ are the subject-specific, Jacobian matrices computed in the testing and training workspaces, respectively. With the intrinsic field defined this way, the forces it produced would be nearly identical to the extrinsic field in the training workspace. Note that the locations of the training and testing workspaces, as well as the **B** matrix, were designed such that the forces in the training workspace were nearly identical, yet nearly orthogonal in the testing workspace (Fig. 3, *C* and *D*). As such, subjects' performance in the testing region when generalizing their newly adapted behavior would clearly dissociate an internal model using extrinsic or intrinsic variables if only one of these variables was used to represent the force field.

To test this idea, subjects participated in three stages of reaching trials nearly identical with the original study (Shadmehr and Mussa-Ivaldi 1994). In the first block, subjects made 250 reaches in the training workspace while the robot generated a null field (zero forces, see below). In 48 randomly chosen trials, the cursor was extinguished at the start of the trial so that subjects reached without visual feedback. After 900 ms the cursor was displayed again, allowing them to bring their hand within the target. Once the block was concluded, the chair was moved to the second position so that the subject's limb was in the configuration appropriate for the testing workspace. The second block consisted of another 250 trials in the null field, again with a random 48 trials in which the cursor was extinguished. These two initial blocks were used to ascertain the subjects' baseline performance in the two workspaces.

The second stage allowed subjects to adapt to the force fields. Subjects were randomly assigned to one of two groups. As in the original study, one group of subjects adapted to the extrinsic field (extrinsic group), while the other group of subjects adapted to the intrinsic field (intrinsic group). Subjects performed two blocks of 500 trials in the force field. Between the blocks subjects were allowed to rest for ∼3 min. Of these 1,000 trials, a random 192 had the cursor extinguished. In half of these trials without visual feedback the robot generated a null field, producing a catch trial. These catch trials allowed us to characterize each subject's ability to predict the perturbing forces while he/she trained.

In the final, third, stage subjects were tested for their ability to generalize in the testing workspace. With minimal delay, the chair was again moved and subjects made another 80 reaches. In all trials the cursor was extinguished. Furthermore, to ensure that each subject made the same number of reaches in each direction, while still allowing for a random ordering of directions, the target sequence was formed with center-out pairs. Thus each subject made 10 reaches in each of the eight directions. Again, as in the original study, four of the subjects (2 from each group) were exposed to a random ordering of extrinsic (27 trials), intrinsic (27 trials), and null (26 trials) fields. For the six remaining subjects (3 from each group) half of these 80 trials were in the extrinsic force field and half in the intrinsic force field.

To examine whether the results we found with the main protocol were robust, we designed two additional variations to the original protocol. In the second version of the experiment (*experiment 2B*), we tested whether vision of one's limb might alter generalization. In a third version (*experiment 2C*), we sought to assess whether a further amount of exposure and time to adapt to the field would change the generalization results. These two additional protocols were identical to the protocol described above, with the following exceptions. In the second protocol, subjects adapted in the training workspace for three blocks, for a total of 1,500 adaptation trials (10 subjects). In the third protocol, a large horizontally oriented plastic board blocked vision of the subjects' arm at all times (10 subjects).

#### Data analysis.

For the analyses presented here only position and force data were used. Cartesian position data of the robot's end point were collected at 400 Hz. These data were used to compute a velocity signal (discretely differentiated and filtered with a 20-Hz, 3rd-order Butterworth filter). To analyze subject adaptation and generalization, a series of metrics were computed. For each trial the MPE, angular error, and normalized path length were computed. The angular error for each trial was computed as the angle between a ray from the subject's starting position to the target and the ray from the same starting position to the point on the path where velocity was maximal. The normalized path length was computed by discretely integrating each trial's path and then dividing by the reaching distance, 10 cm.

In addition to these metrics, correlations and the metric ρ (defined in Shadmehr and Mussa-Ivaldi 1994 but with a typographical error corrected in the Appendix of Caithness et al. 2004) were computed. To do this, each trial was translated to the origin (in *x* and *y*) and temporally aligned to the point where the velocity first reached the value of 0.1 m/s. On a subject-by-subject basis, the last 200 trials of the baseline blocks were sorted according to reach direction and feedback condition (cursor on or extinguished) and averaged; the result was an average reach to each target, for each feedback condition, for each subject. With these subject- and direction-specific reach trajectories, correlations with average reaches, and ρ, could be computed. The correlations were computed using position and velocity data, comprising a window from ∼125 ms before and 750 ms after the alignment point. As defined elsewhere, ρ was computed with the same data window, but using a running windowed average of the inner product between each trial's velocity and the corresponding average velocity trace from baseline.

Tests for significance were performed with paired *t*-tests as well as repeated-measures ANOVA tests. Significance levels were set to 0.05.

#### Experimental apparatus.

The robotic apparatus used is an updated version of that used in Shadmehr and Mussa-Ivaldi (1994). The manipulandum has two torque motors (PMI Motor Technologies model JR24M4CH) used to generate forces at the handle. Two position encoders were used to record the angular position of the two robotic joints with a resolution exceeding 20 arcsec of rotation (Teledyne Gurley model 25/045-NB17-TA-PPA-QAR1S). The position, velocity, and acceleration of the handle were derived from these two signals. End-point forces and torques were monitored with a six degree-of-freedom load cell fixed to the handle of the robot (Assurance Technologies model F/T Gamma 30/100). Software written for this experiment was run in MATLAB'S real-time XPC platform at 400 Hz. On each sample the subject's kinematics were calculated and used to compute any end-point forces (extrinsic, intrinsic, or null field). A model of the robot (τ_robot_) was used to partially cancel its inertial mechanics. In addition, a low-gain force-feedback loop was also used to help cancel the difference between the forces measured at the robot handle, *F*_measured_, and those commanded. The gains on the inertial and force-feedback torques/forces were chosen to keep the robot stable while maximizing the fidelity of the rendered forces. The commanded torques were as follows:

$$\tau =J^{T}F+0.3\tau_{\text{robot}}+0.4J^{T}\left(F-F_{\text{measured}}\right)$$

## RESULTS

### Experiment 1

Subjects performed an experiment to examine the coordinate system used to represent newly adapted dynamics. In the initial preexposure stage, we quantified baseline performance while subjects moved in a null field. In the subsequent exposure stage, subjects adapted to a velocity-dependent force field. In the final generalization stage, subjects continued to move in the force field and occasional force channel trials were used to probe their generalization behavior. These generalization trials, which involved changes in shoulder and elbow angles as well as hand orientation, were designed to test for three possible coordinate frames: extrinsic, intrinsic, and object based.

#### Force-field adaptation.

**Fig. 4.**
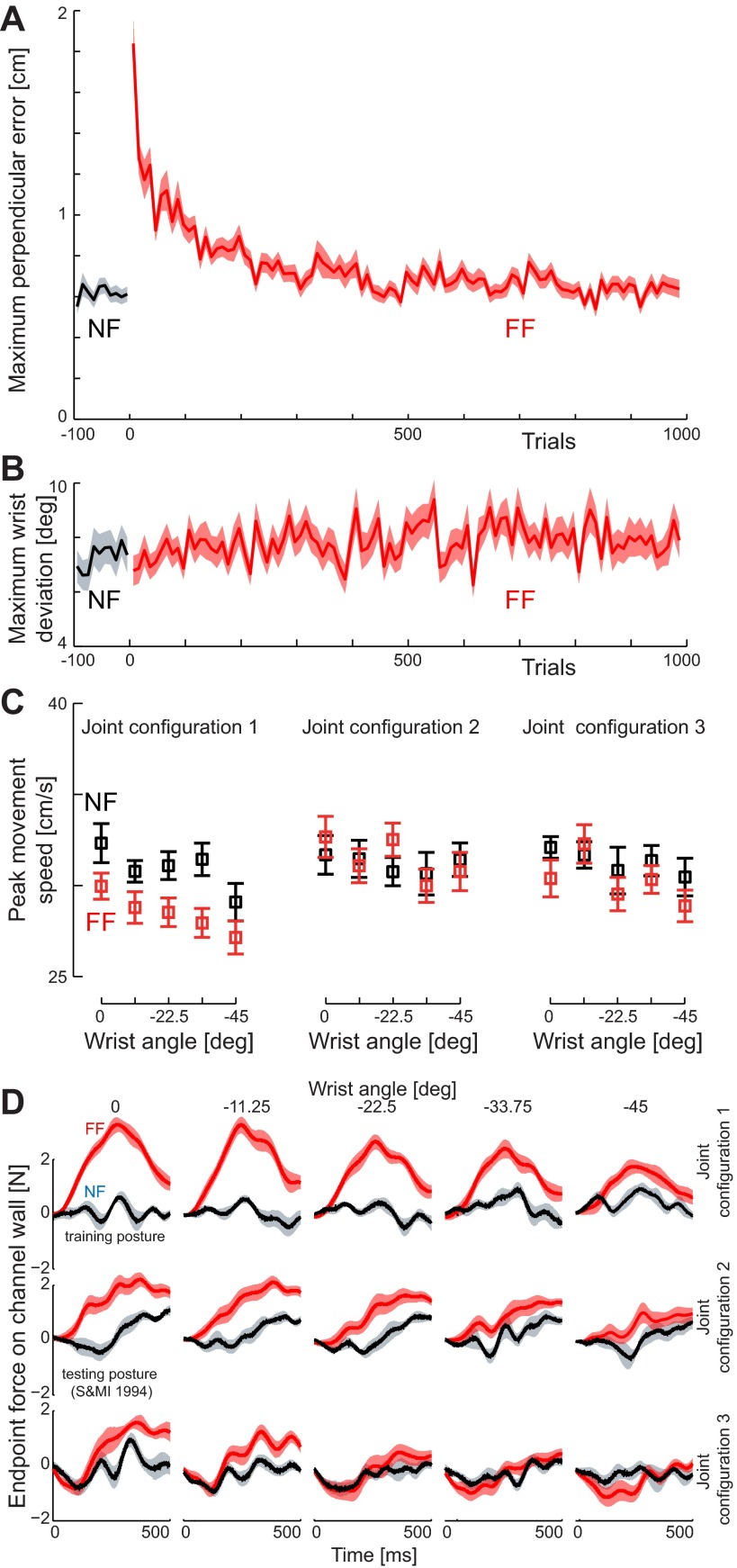
Learning of the force field dynamics in *experiment 1*. *A*: maximum perpendicular error (MPE) is plotted against the trial number for training movements in the initial null field (NF, black) and in the skew viscous force field (FF, red). The mean (solid line) and SE (shaded region) are shown across all subjects. *B*: mean peak wrist rotation (±SE) across all subjects is shown for the movements in the null field (black) and skew viscous force field (red) during the training movements. Note that no increase in wrist motion is seen when the force field is introduced. *C*: peak speed in the channel trials at each of the 15 test postures. The mean peak speed (±SE) across all subjects is shown for the movements in the null field (black) and skew viscous force field (red) during the test movements. Results are plotted as a function of the change in wrist angle rather than absolute hand orientation. *D*: force generalization. The mean (±SE) force trace during the channel movements is shown for movements in both prelearning (black) and late exposure (red) in the test movement trials.

In the preexposure stage, subjects made movements in the null field with a single hand orientation in the training workspace. They did so with little deviation from a straight-line movement, such that the MPE was small (Fig. 4*A*). In the exposure stage, when the velocity-dependent force field was unexpectedly applied (training workspace with a single hand orientation), subjects' trajectories were initially perturbed by the forces (large increase in MPE; Fig. 4*A*). During this training period subjects were able to learn to make straighter movements to the target with the MPE reducing over the trials, until by the end of the experiment MPE was close to the level of the preexposure movements (Fig. 4*A*).

The values of MPE in the training workspace with a single hand orientation were investigated with an ANOVA with three conditions (final 20 null-field movements, initial 20 force-field movements, and final 20 force-field movements). After an initial significant main effect of condition (*F*_2,16_ = 9.264; *P* = 0.002), the differences across the three parts of the experiment were examined with Tukey's honestly significant difference (HSD) post hoc tests. Initial movements in the force field were significantly larger than those in the null field (*P* = 0.001), indicating that the introduction of the force field perturbed the subjects' movements. However, subjects were able to adapt to the force field as the MPE reduced significantly by the end of the force field trials (*P* = 0.001), with no significant difference between the MPE during the final preexposure trials and those in the final exposure trials (*P* = 0.993).

Previous work on force-field adaptation has assumed that subjects maintain a constant wrist angle during movements with the force field (Malfait et al. 2002, 2005; Shadmehr and Moussavi 2000; Shadmehr and Mussa-Ivaldi 1994) and therefore play a negligible role in the dynamics of movement. To assess wrist movements we examined the maximum angular deviation of the wrist, comparing the final 20 preexposure movements, the initial 20 exposure movements, and the final 20 exposure movements. No differences among these sets of trials were observed (*F*_2,16_ = 0.586; *P* = 0.57), and, as shown in Fig. 4*B*, the maximum deviation of the wrist was quite small throughout.

In test trials, the force channel generates lateral force that matches the lateral force that subjects expect (and thus generate). However, the channel does not match the expected forces along the channel, and it is possible that this mismatch might significantly alter movement speed. To assess this possibility, we examined the peak speed of the hand, in the direction of the target, for each of the 15 generalization movements (Fig. 4*C*). An ANOVA, which included a main effect of generalization movement (15 levels), showed a significant difference in hand speed movement between the preexposure and generalization stages (*F*_1,8_ = 16.29; *P* = 0.004, with slowing in the field), but importantly there was no significant interaction between the stages and generalization movement (*F*_14,112_ = 1.09; *P* = 0.37). The movement speed was therefore unaffected by any predictive force compensation.

#### Generalization and model predictions.

**Fig. 5.**
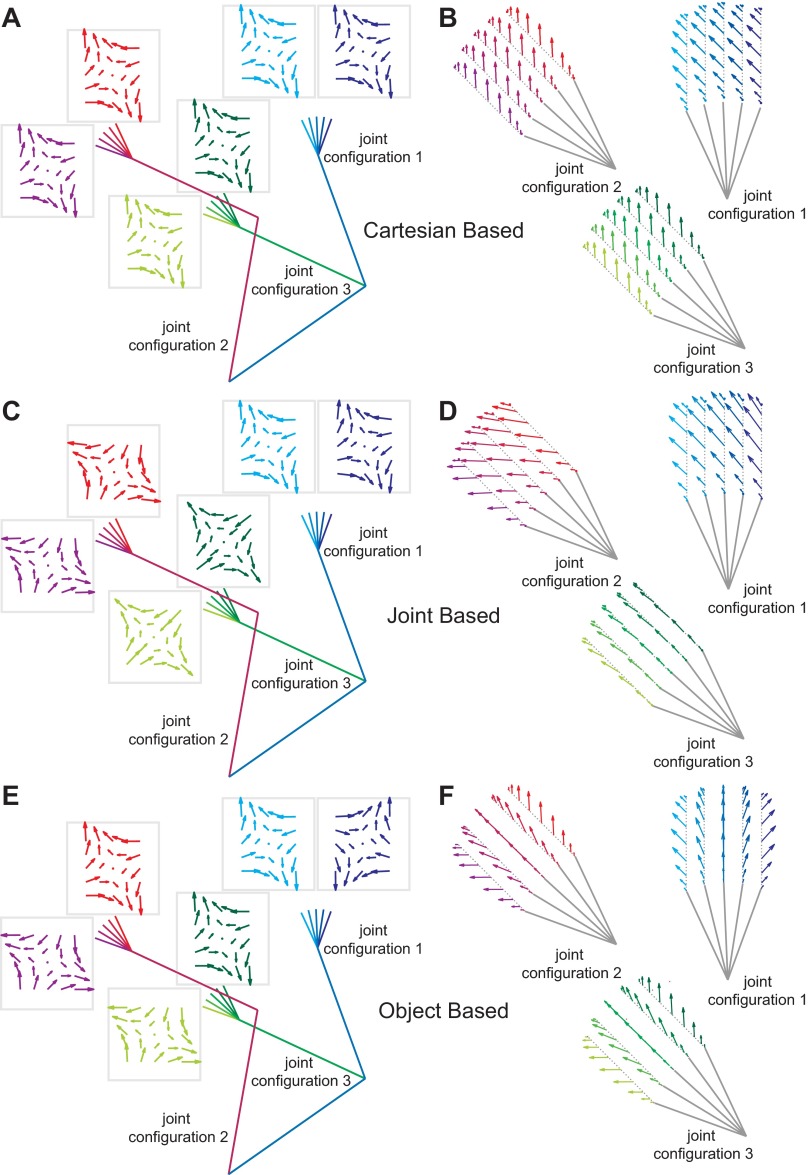
Predictions of the 3 models for *experiment 1*. *A*: in a Cartesian-based generalization the force field remains constant in Cartesian space despite the change in limb geometry. The force field plots show force vectors as a function of hand velocity (as in Fig. 3*D*), with each of the 6 fields corresponding to the 3 joint configurations and the 2 extreme hand orientations (wrist flexion and extension) for these configurations. The color codes of the force fields are matched to their corresponding posture. *B*: the forces expected for Cartesian-based generalization are shown for a minimum jerk movement for the 5 hand orientations in the 3 configurations. Each configuration had 1 movement direction selected to be maximally informative in distinguishing between the 3 coordinate systems. In this case, the forces do not depend on the hand orientation or joint configuration but only on movement direction. *C*: for joint-based generalization the expected force field changes for each joint configuration. Because of the noninvertibility of the Jacobian for the 3-joint model, we use here the model based on the virtual segment (see materials and methods), which leads to very similar force predictions. *D*: joint-based generalization (virtual segment) predicts a different pattern of generalization for each joint configuration that is also different from the Cartesian generalization (*B*). The hand orientation affects the direction of the expected forces slightly. *E*: for object-based generalization, the expected force fields should rotate with the hand orientation. Therefore, as the hand orientation rotates by 45° (through any combination of wrist, elbow, or shoulder rotation), so does the expected force field. *F*: the forces expected for object-based generalization vary with hand orientation and are strikingly different from Cartesian (*B*) or joint-based (*D*) generalization.

In both the preexposure and generalization stages of the experiment, on random trials subjects were asked to produce 1 of 15 generalization movements involving different shoulder, elbow, and wrist postures. In these movements the robotic interface constrained the hand to a channel from the start of the movement to the target (channel trials). Figure 5 shows the predictions for generalization in different possible coordinate frames for each of these 15 test movements. Note that the predicted force fields (Fig. 5, *A*, *C*, and *E*) and forces (Fig. 5, *B*, *D*, and *F*) are displayed in Cartesian coordinates to allow comparison among the predictions. If generalization occurs in Cartesian coordinates, then the expected force field will be independent of joint configuration and hand orientation (Fig. 5, *A* and *B*). Of course, the end-point forces will depend on the direction of movement, leading to different forces for *joint configuration 1* compared with *joint configurations 2* and *3*. If generalization occurs in joint-based coordinates, the expected force field will vary substantially across joint configurations and slightly with the change in the hand orientation (Fig. 5, *C* and *D*). Finally, if generalization occurs in object-based coordinates, the expected force field will change significantly with the orientation of the shoulder, elbow, or wrist, as all three angles affect the orientation of the hand in external space (Fig. 5, *E* and *F*). Therefore, the extrinsic, intrinsic, and object-based hypotheses make strikingly different predictions about the force compensation that would be expected for each testing posture (Fig. 5).

In the preexposure stage, hand forces perpendicular to the movement direction (i.e., against the channel wall) during test movements were close to zero (Fig. 4*D*). As expected, in the generalization stage that followed exposure to the force field, substantial perpendicular hand forces were observed during generalization movements. The mean perpendicular force 50–450 ms after movement initiation in these movements was significantly larger in the generalization stage than in the preexposure stage (*F*_1,8_ = 38.979; *P* < 0.001). As illustrated in Fig. 4*D*, the largest forces were produced in the training posture (i.e., *joint configuration 1* with a wrist orientation of 0°) and force tended to decrease with changes in both joint configuration and wrist orientation. A 3 × 5 (configuration × wrist orientation) ANOVA revealed main effects of both configuration (*F*_2,16_ = 19.008; *P* < 0.001) and wrist orientation (*F*_4,32_ = 11.294; *P* < 0.001) but no significant interaction (*F*_8,64_ = 0.470; *P* = 0.873).

**Fig. 6.**
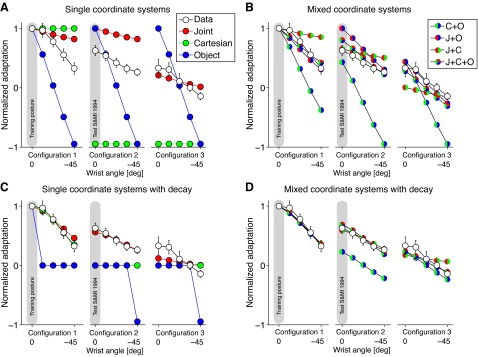
Adaptation on generalization trials and model fits. *A*: mean adaptation (±SE across subjects) as function of generalization postures (Data): model fits for the joint-based, Cartesian-based, and object-based coordinate system models. All predictions and the subjects' data are normalized to 1 at the training posture. *B*: fits of the mixture of coordinate system models. The weighting of the components was required to sum to 1 *C*: fits of the single-coordinate system models with decay. *D*: the first of the mixture of coordinate system models with decay.

The force on channel trials was also compared to the force predicted by the three models (Fig. 6*A*). The extrinsic Cartesian-based model (Fig. 6*A*) predicts high positive levels of force across all five movements in *joint configuration 1* and high negative levels of force across all movements in *joint configurations 2* and *3*. These results are clearly inconsistent with the idea that generalization occurs purely in Cartesian coordinates. The joint-based model (Fig. 6*A*) predicts high positive levels of force for *joint configurations 1* and *2* and little force for *joint configuration 3*, with small variations with hand orientation in all three cases. This model, like the Cartesian model, fails to fully capture the generalization forces. Moreover, it does not predict the data well even when considering the test posture (i.e., *configuration 2*) used by Shadmehr and Mussa-Ivaldi (1994). Specifically, for this particular posture, the perpendicular force is approximately half the level produced at the training configuration. Finally, the object-based model (Fig. 6*A*) predicts that the force should decrease as a function of decreasing wrist angle for all three joint configurations. While this model captures the modulation in force with wrist angle, the overall fit is visibly poor.

#### Mixture of coordinate systems.

**Fig. 7.**
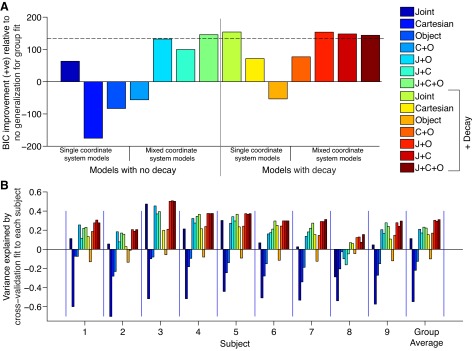
Model comparisons. *A*: Bayesian information criterion (BIC) improvement for each of the models relative to no generalization (that is, a model in which the force is zero at all the generalization postures). Dashed line shows the cutoff for models that are not considered to be distinguishable in terms of their performance from the best model. *B*: leave-one-out cross-validation analysis. For each subject we show the variance explained for each model when fit to the average of the remaining subjects' data. The final column shows the average of the variance explained across subjects. The model color code is as in *A*.

We examined whether a mixture of coordinate systems could account for the pattern of generalization seen in our subjects. For each subject and generalization movement we calculated the mean perpendicular forces and fit these concatenated data with different linear combinations of the predictions of the three coordinate systems (Fig. 6*B*). We used the BIC to compare all one-, two-, and three-coordinate system models. Figure 7*A* shows the BIC relative to no generalization (that is, a model in which the force is zero at all the generalization postures). This shows that of the single-coordinate system models the joint-based model is the best and, in fact, the Cartesian and object-based models perform worse than the baseline model with no generalization. All mixture models that included predictions from the joint-based model (see J+O, J+C, J+C+O in Fig. 7*A*) provided significantly better fits (i.e., the difference in BIC was between 36 and 82 units) than the single-coordinate joint-based model, with the mixture of three coordinates system (J+C+O) performing best.

The cross-validation analysis allows us to compare each subject's performance to the predictions obtained from the fits to the remainder of the subjects (Fig. 7*B*). The plots show the variance explained by each model as well as the group average (the variance explained can go negative for models that have worse predictive performance than the overall mean perpendicular force). This shows that although there is variability across subjects, in general the mixture models perform better than any single-coordinate system model.

#### Decaying generalization models.

We also examined decaying generalization models in which the force on generalization trials was assumed to be represented within a single coordinate system but with a magnitude that decays with distance—in that coordinate system—from the training posture (Fig. 6*C*). The BIC analysis (Fig. 7*A*) shows that the joint-based decay model performs well, and significantly better than the Cartesian or object-based decay models. We also fit mixture models incorporating two- and three-coordinate system models with decay (Fig. 6*D*). The BIC analysis showed that the best model was the joint-based decay model, which was marginally better than the J+O decay mixture model (0.4 BIC units difference). The dashed line in Fig. 7*A* shows the cutoff for models that are not considered to be distinguishable in terms of their performance from the best model (i.e., 20 BIC units from the maximum, which corresponds to a Bayes factor of 10). Therefore, the full mixture model without decay, the joint-based decay model, and three mixture models with decay (J+O, J+C, and J+C+O) all provide equivalent fits to the data. Similar results are seen in the individual subject cross-validation (Fig. 7*B*). The best parameters of the models, degrees of freedom, and BICs are provided in Table 1.

**Table 1.** Model fits to subject data for experiment 1

Model	*k*_j_	*k*_c_	*k*_o_	*d*_j_, rad^−2^	*d*_c_, m^−2^	*d*_o_, rad^−2^	dof	BIC	BIC Improvement over No Generalization
No-decay models									
Joint	1	—	—	—	—	—	0	−212.0	63.4
Cartesian	—	1	—	—	—	—	0	26.5	−175.2
Object	—	—	1	—	—	—	0	−65.6	−83.1
C+O	—	0.29	0.71	—	—	—	1	−92.5	−56.2
J+O	0.71	—	0.29	—	—	—	1	−280.9	132.2
J+C	0.82	0.18	—	—	—	—	1	−248.5	99.8
J+O+C	0.66	0.10	0.24	—	—	—	2	−294.3	145.6
Decay models									
Joint	1	—	—	0.93	—	—	1	−302.7	154.0
Cartesian	—	1	—	—	365.88	—	1	−220.2	71.5
Object	—	—	1	—	—	3.01	1	−101.0	−47.7
C+O	—	1	0	—	365.93	N/A	3	−210.6	61.9
J+O	0.88	—	0.12	0.71	—	0	3	−302.3	153.6
J+C	1	0	—	0.93	N/A	—	3	−293.0	144.3
J+O+C	0.88	0	0.12	0.71	N/A	0	5	−292.6	143.9

The *k* values are the mixture parameters for the individual coordinate system predictions, which are constrained to sum to 1. The *d* values are the decay parameters associated with distance from the training configuration in the 3 different coordinate systems (with the units of distance in radians for angles and meters for Cartesian distance). The final 3 columns are the degrees of freedom (dof) of the model, the Bayesian information criterion (BIC), and the BIC improvement compared with the no-generalization model. Dashes are used to indicate parameters that are not fit and are set to zero for the particular model. N/A refers to fitted parameters that do not affect the model predictions, as the corresponding mixing parameter has a fitted value of zero.

### Experiment 2

#### Behavioral observations.

The extrinsic/intrinsic dichotomy (Shadmehr and Mussa-Ivaldi 1994), according to which generalization takes place in one of the two coordinate systems, has been central to the study of generalization. Since numerous studies have presented conflicting findings on the coordinate frames of generalization, we have attempted to carefully reproduce the original experiment. Subjects adapted to one of two force fields, either extrinsically or intrinsically defined, while making point-to-point reaching movements to pseudorandomly generated targets. Their ability to make reaches in a new area of their workspace was then probed by exposing all of the subjects to these two force fields. If subjects adapted by learning to predict the perturbing forces in intrinsic coordinates, they would generalize well in the intrinsic field, and vice versa for the extrinsic field. Since our experiment is a reexamination of a previous, well-known study, we begin by providing a qualitative description of our findings and where they differ from the original study.

**Fig. 8.**
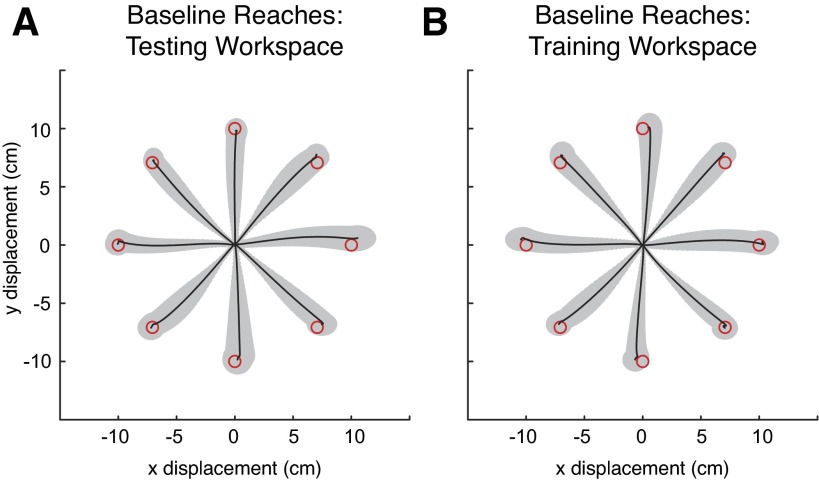
Average hand paths during baseline null field reaching in *experiment 2*. For each subject, the last 200 trials were processed accordingly: the starting location for each hand path was translated to the origin (in *x* and *y*) and then temporally aligned to the point in time where the velocity first reached the value of 0.1 m/s. These paths were sorted according to reach direction and feedback condition. Displayed are the across-subject averages and SDs of reaches made without visual feedback in the testing workspace (*A*) and the training workspace (*B*) along with the locations of the targets (drawn to scale).

To characterize trajectories in the absence of perturbations, all subjects initially performed a block of 250 trials in the training workspace and then a block of 250 trials in the testing workspace. All 500 of these reaches were in a null field and included random trials within which vision of the cursor was absent. Early reaches in both workspaces were curved, but over the course of several dozen trials reaches became straight (Fig. 8 depicts late reaches during baseline). Unsurprisingly, there were no qualitative differences between reaching behaviors in the training and testing workspaces.

**Fig. 9.**
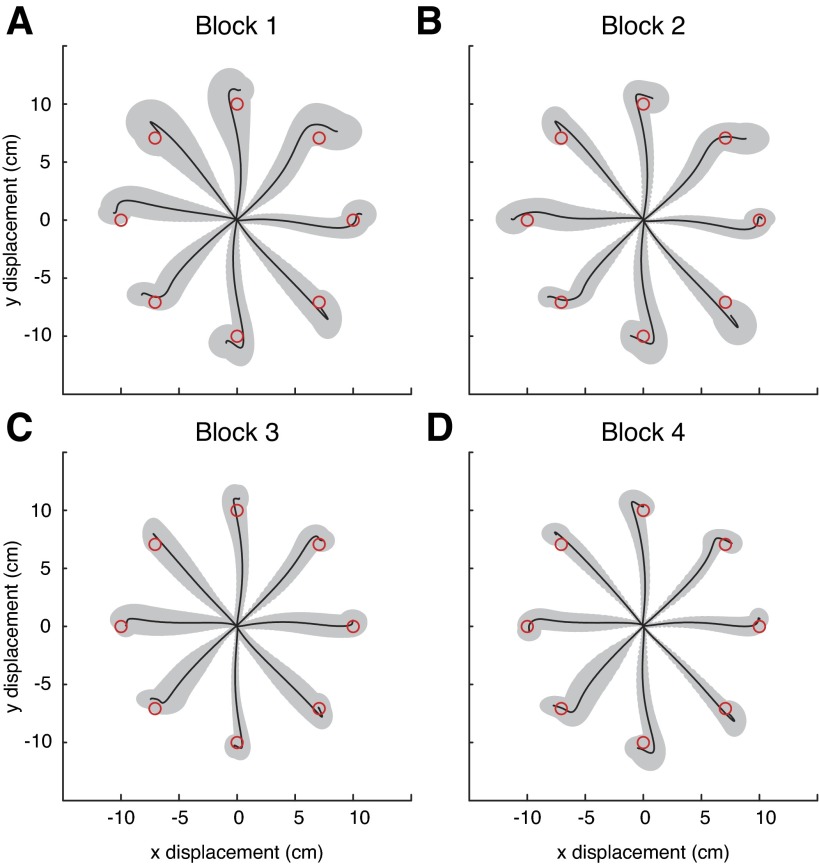
Average hand paths during adaptation trials in *experiment 2*. Similar to Fig. 8, using all 1,000 trials of adaptation, paths were translated to the origin, temporally aligned, and sorted according to target and feedback condition and force field. Displayed are the across-subject averages for no-vision, force field trials during the first (*A*), second (*B*), third (*C*), and last (*D*) 250 trials of adaptation.

After the baseline blocks we collected data during adaptation to the force fields. Subjects performed two blocks of 500 trials each, with a 3-min rest period in between. Five subjects were exposed to the extrinsic force field (extrinsic group, *Eq. 1*), while another five subjects were exposed to the intrinsic force field (intrinsic group, *Eq. 2*). The intrinsic field was designed such that the forces produced were nearly identical to those of the extrinsic field in this training workspace. The force fields perturbed subjects based on the direction and speed of their movement (for the subjects in the intrinsic group, there was slight dependence on their limb's posture as well). The force fields, having a stable and an unstable axis, tended to accelerate reaches in some directions, impede reaches in other directions, and laterally perturb reaches in intermediate directions. As is typical, with continued exposure to the force field reaching paths became less curved, their ability to land on target in the specified time improved, and in general the influence of the force field became less apparent (Fig. 9). This was true for both reaches made with vision and those made without.

**Fig. 10.**
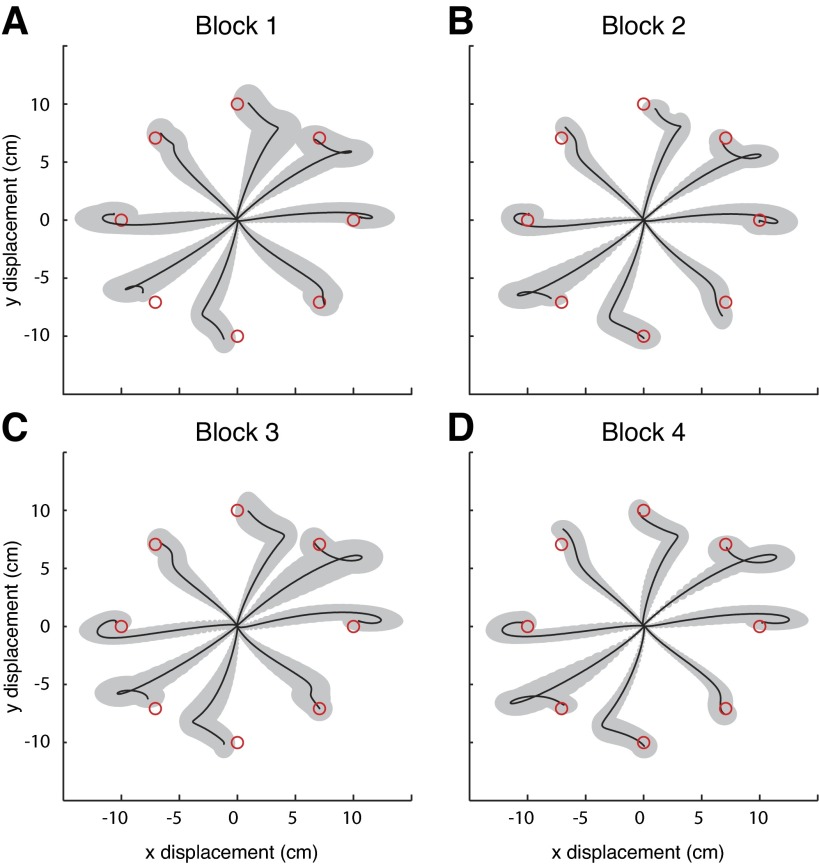
Average hand paths during no-vision, null field, catch trials in *experiment 2*. Similar to Fig. 9, paths are translated to origin, temporally aligned, and sorted to target. Displayed are the across-subject averages for the catch trials in the first (*A*), second (*B*), third (*C*), and last (*D*) 250 trials of adaptation.

To determine whether the observed changes were merely the result of cocontraction, or rather a predictive change in the motor commands, catch trials were randomly interleaved with the force field trials. In these catch trials, vision of the cursor was absent and a null force field was present. In these trials, reaches deviated in the opposite direction to those in early learning, as if perturbed by a mirror symmetrical force field (Fig. 10). The effects of these catch trials grew over time, and their paths became more regular (compare the standard deviations between Fig. 10, *A* and *C*), suggesting that the subjects improved in predicting the effects of the force field.

**Fig. 11.**
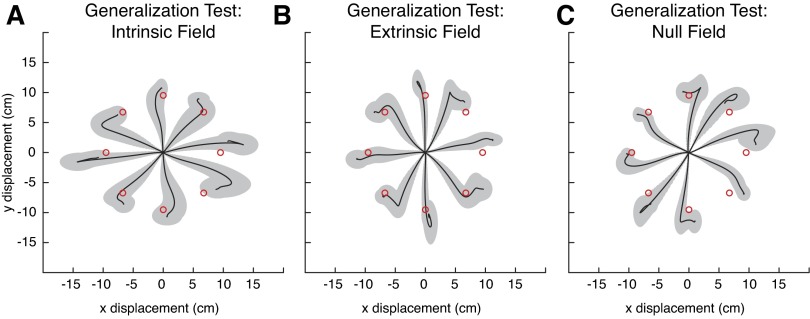
Average hand paths during generalization trials in the testing workspace in *experiment 2*. For each subject (regardless of training group), all generalization reaches are translated to origin, temporally aligned, and sorted according to target and force field. Displayed are the across-subject averages for the trials with the intrinsically defined field (10 subjects; *A*), the extrinsically defined field (10 subjects; *B*), and the null field (4 subjects; *C*).

Finally, we probed the ability of subjects to generalize. With their arm again in the testing workspace, they made 80 reaches while the field switched randomly between the extrinsic, intrinsic, or null fields. All trials were made without vision of the cursor (see materials and methods). As in the initial trials of the first adaptation block, subjects' reaches were clearly perturbed by the fields. In contrast with the original study (Shadmehr and Mussa-Ivaldi 1994), it was not evident that subjects could compensate for the intrinsic force field any better than the extrinsic field; indeed, it was not evident that they were even better at making reaches in either field relative to the null field (Fig. 11). Subjects showed no clear evidence of generalizing better in one field over another.

Therefore, in terms of the basic learning of the force field, our results agree with the original study. Subjects adapted to force fields, and their trajectories became straighter. This was not just due to cocontraction, since there were predictive components leading to aftereffects. This adaptation also affected generalization of movements to a test region. However, we were unable to replicate the original generalization results. We found no signs of a clear generalization pattern in intrinsic coordinates. This surprising finding, and the behavioral phenomena leading up to it, are analyzed below.

#### Behavioral analysis.

We began by analyzing reaches made during the baseline blocks. We averaged over the last 200 baseline trials after translating all the reaches to a common origin and grouping them according to the eight different reaching directions. Reaches were very typical in both workspaces, with and without visual feedback, with straight paths and approximately bell-shaped velocity profiles. To quantify the similarity between the two workspaces, correlations of the average reaches (both position and velocity) were computed. Correlations across the training and testing workspaces were very high, ∼1.0 for both reaches made with vision (data not shown) and those made without (Fig. 8).

**Fig. 12.**
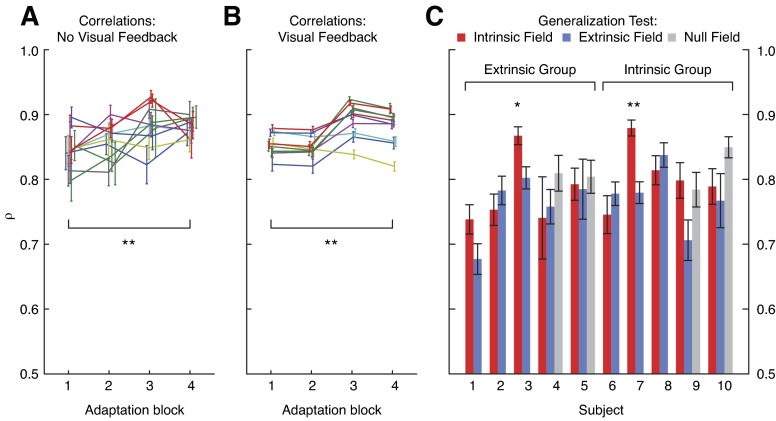
Individual subject performance during generalization trials in *experiment 2* as measured with correlation ρ (see materials and methods). *A*: average correlations during adaptation trials without visual feedback. *B*: average correlations during adaptation trials with visual feedback. *C*: average correlations during the generalization block. Trials in intrinsic, extrinsic, and null fields are grouped separately for each subject. All error bars are SEs. Statistical significance: *P < 0.05, **P < 0.01.

To assess subject performance during adaptation, we first performed the same analysis as in the original study. The reaching trajectories during adaptation were compared with their corresponding averages during the baseline in the same workspace. To do this we used the correlation defined in Shadmehr and Mussa-Ivaldi (1994), ρ (see materials and methods), which is a running average of the inner product of velocity traces. The average velocity traces during baseline (in each of the 8 directions, during the last 200 trials without vision) were compared with their respective averages during the first, second, third, and last 250 trials of adaptation (again, separated by direction and using only those reaches made without vision). With this measure we can quantify how similar reaches during adaptation are to reaches during baseline. The correlation ρ began relatively high, with an across-subject average of 0.85 during the first 250 trials (Fig. 12*A*). All subjects showed an improvement in this correlation as the trials progressed, and the across-subject average during the final 250 trials, 0.88, was significantly larger (paired *t*-test, *P* < 0.01). Similarly, we also compared ρ for the trials with visual feedback (here the trials were compared against their averages made with visual feedback in the null field). Despite two subjects who showed a small decrease, the across-subject average increased from 0.85 to 0.88, a significant improvement (*P* < 0.01; Fig. 12*B*). These findings corroborate the qualitative observation that over time subjects' reaches tended to become similar to those made during baseline.

Following the original study's lead, we then compared generalization in the extrinsic and intrinsic reaches using the correlation ρ. In contrast with the original study, there was no clear difference between extrinsic, intrinsic, and even null fields (Fig. 12*C*). On the whole, we found that subject performance was roughly similar in both the extrinsic and intrinsic fields. The across-subject average for the extrinsic trials was 0.77, whereas the across-subject average in the intrinsic field was 0.78. In fact, even performance in the null fields was qualitatively similar when examined with this metric (an average of 0.81). Whereas in the original study subjects' performance when generalizing in the intrinsic field was clearly better than in the extrinsic field, we found that only two subjects displayed significant improvements in the intrinsic field (adjusted for 10 comparisons). This qualitative discrepancy with the original study merited further scrutiny.

To ask which factors affect generalization performance as quantified by ρ, we ran additional statistical tests. We computed a repeated-measures ANOVA to test for an effect of the group type (either extrinsic or intrinsic) or the three different fields during generalization. There was no significant effect of group (*F*_1,20_ = 0.937; *P* = 0.345), and while clearly affecting trajectories, the force fields had no significant effect on ρ (*F*_2,20_ = 1.529; *P* = 0.241). This might have been expected, since performance in the null field was different (and indeed often better—in 3 of the 4 subjects that experienced the null field, their scores were best in the null trials) from performance in either the extrinsic or intrinsic fields (Fig. 12*C*). This metric, ρ, relies on multiple free parameters (e.g., the number of data points, how the velocity traces are aligned, etc.), and it is not clear how well it quantifies generalization performance. We thus wanted to be careful not to prematurely conclude that our findings were distinct from the original study. Similarly, given that ρ is merely one of a number of possible ways to quantify behavior, we attempted to quantify subjects' performance in multiple additional manners to search for a distinction between extrinsic and intrinsic generalization.

For additional metrics, we choose the commonly used maximum perpendicular deviation from a straight reach to the target, the angular deviation from a straight reach, and the normalized path length. These three metrics have the benefit of not relying on any experiment-specific parameters such as sampling rate or how different reaches are aligned. Finally, for comparison with ρ, we also examined the correlation between trajectories and their corresponding baseline reaches. As in the original study, we used these four metrics to examine subjects' reaches, and to look for clear evidence that the subjects performed better in one of the two generalization fields.

**Fig. 13.**
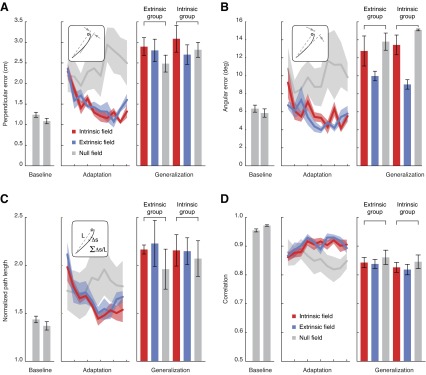
Subject performance during generalization trials in *experiment 2*: performance metrics of maximum perpendicular deviation from a straight reach to the target (*A*), angular deviation from a straight reach (*B*), normalized path length (*C*), and correlation with position and velocity of baseline reaches (*D*). *Left*: across-subject average measures during baseline (null field), averaged across extrinsic and intrinsic groups (gray bars show training workspace, then testing workspace; error bars are SEs). *Center*: 250-trial bins of adaptation for both groups along with the null field catch trials (averaged across both groups). *Right*: across-subject averages for generalization trials (error bars are SEs).

We first describe the results obtained analyzing the perpendicular error (Fig. 13*A*); the analyses performed with the other metrics were equivalent. The perpendicular error was averaged across all reaches without visual feedback in the training and testing workspaces (Fig. 13*A*, *left*; error bars are SEs). The average error in both workspaces was relatively small, ∼1–1.25 cm, with an error in the testing workspace roughly a quarter centimeter smaller (though not significantly so, paired *t*-test, *P* = 0.065). The perpendicular error during the adaptation and catch trials in the absence of visual feedback was binned into 10-trial intervals (Fig. 13*A*, *center*). Relative to baseline, the perpendicular error increased by roughly 1 cm in the first bin of adaptation. There was also a similar increase in the first 10 catch trials. However, as the trials continued, the perpendicular error during adaptation trials progressively decreased, while the catch trial errors increased. By the end of the adaptation blocks, the perpendicular error had significantly decreased and was statistically indistinguishable from baseline errors (*P* = 0.083). The catch trial errors, on the other hand, were now relatively large. These results further verified that subjects were able to adapt to the force fields, and did so in a manner consistent with predictive behavior.

During generalization, the perpendicular error was grouped according to the field experienced (extrinsic, intrinsic, or null) and the training group (extrinsic or intrinsic). On the whole, the errors during generalization were large, even larger than those measured during catch trials (Fig. 13*A*, *right*; error bars are SEs). Moreover, the error was relatively consistent across conditions, and not a single one was significantly better than the others. As a further comparison, we performed a repeated-measures ANOVA to test for the effects of force field and training group, but neither had a significant effect (*F*_2,20_ = 0.768, *P* = 0.477 and *F*_1,20_ =0.190, *P* = 0.668, respectively). Identical analyses were performed with the angular errors, normalized path lengths, and correlations (Fig. 13, *B*, *C*, and *D*, respectively). The only statistically significant effect found was for the angular errors, where there was an effect for the force field (*F*_2,20_ = 9.937; *P* = 0.001). In contrast with the original finding, a post hoc test determined that angular errors in the extrinsic field were significantly smaller than in the other fields. Taken together, there was no clear evidence for an ability to generalize better in the extrinsic or intrinsic fields.

Given the importance of the original finding, and the implications of our failure to replicate it, we ran another two groups of subjects with slight variations to the original protocol. In the second experiment (*experiment 2B*), 10 subjects performed the same protocol described above, but without vision of their limb. This experiment examined whether vision of the limb biased performance to either intrinsic or extrinsic coordinates. In a third experiment (*experiment 2C*), 10 subjects trained with the force fields for an extended period of 3 blocks of 500 trials each. This experiment examined whether the generalization patterns we observed were due to the amount of adaptation or the lack thereof. These two additional experiments would help establish whether our findings were robust, or rather somehow contingent on the details of our protocol and experimental apparatus.

In *experiment 2B*, the lack of vision of the arm had little effect on subjects' overall performance. Just as before, subjects initially generated large errors but by the end of the adaptation blocks showed significant improvements (data not shown). As before, ANOVAs were performed to quantify the effects of the force field and training group for the four metrics. There was no effect of force field for any of the five metrics (*P* > 0.05). There was an effect for training group with path lengths (*F*_1,20_ = 6.223; *P* = 0.022); however, the intrinsic errors were larger. Finally, there was an effect of training group for the correlations (*F*_1,20_ = 4.524; *P* = 0.046), where subjects in the intrinsic group had smaller correlations with baseline. Here again, the results presented no clear evidence for generalization in one coordinate system over the other.

In *experiment 2C*, the overall behavioral results and analysis showed little change with the added 500 adaptation trials. Subjects adapted to the force fields but showed no clear ability to generalize better in either field. Across all five metrics ANOVAs found no significant effects for the training groups but a single effect for the force fields in ρ (*F*_2,20_ = 3.928; *P* = 0.035). A Tukey post hoc test indicated that the correlations were significantly smaller in the extrinsic field, yet there was no significant difference between the reaches in the intrinsic and null fields. These findings, taken together with the results of *experiments 2B* and *2A*, very clearly argue that subjects do not generalize in either an exclusively extrinsic or intrinsic pattern.

## DISCUSSION

We performed two experiments reexamining the issue of the coordinate frames used when learning novel dynamics in the context of reaching movements. The first experiment was designed to test between three possible accounts of the original findings of Shadmehr and Mussa-Ivaldi (1994). Specifically, we sought to test whether novel dynamics are encoded in intrinsic joint-based or extrinsic coordinates, as suggested by Shadmehr and Mussa-Ivaldi, or object-centered coordinates linked to the grasped handle. Although subjects clearly adapted to the force fields, their behavior during generalization did not conform to a simple interpretation in terms of encoding in any one of these three categorical coordinate frames. Notably, we did not obtain clear evidence supporting previous work suggesting that dynamics are encoded in intrinsic coordinates (Malfait et al. 2002; Shadmehr and Mussa-Ivaldi 1994). The second experiment attempted to reproduce the original protocol of Shadmehr and Mussa-Ivaldi. Here again, we found that although subjects clearly adapted to the force fields, their ability to generalize was modest at best and did not appear to favor force field encoding in either intrinsic or extrinsic coordinates.

Our experiments were designed so that subjects would exhibit relatively unambiguous evidence if they generalized using a single coordinate frame that generalized globally. Despite having adapted to the experimental perturbations, there was no evidence for the encoding of this information in a single coordinate frame. This suggests that dynamics may be represented using a combination of coordinate frames or some local representation that decays with distance from the training location. To assess these possibilities, we used data from *experiment 1* to evaluate one-, two-, and three-component mixture of coordinate system models, all with and without spatial decay. We found that the data could be equally well fit with the three-component mixture model—combining joint-based, Cartesian, and object-centered coordinate frames—without decay, the joint-based decay model, and three different mixture models with decay (Fig. 7). Given the similarity in fits of these models, we have not provided clear support for any one of these models. However, our results clearly show that all five of these models perform better than a joint-based model without decay. In *experiment 2*, we found that despite having adapted to the experimental perturbations subjects' motor performance in the generalization area was poor, at times no better than naive performance. Here again the evidence denied a simple interpretation in terms of a single coordinate frame that generalized globally.

One possible interpretation for our collective results is that newly adapted knowledge related to motor behavior is represented locally with limited and graded generalization. If the nervous system adapted by estimating a parameterized model, such as the matrix of terms that defines a velocity-dependent force field, generalization should be complete (essentially equal in performance in all areas of the workspace). On the other hand, if the nervous system only learns locally around the input-output pairs specifically experienced, such as states visited and forces/commands produced, then generalization would decrease as the change in context (e.g., limb configuration) increases. There is considerable evidence that sensorimotor adaptation is a form of local learning that does not generalize to very different circumstances (Burgess et al. 2007; Donchin et al. 2003; Gandolfo et al. 1996; Krakauer et al. 2000; Lackner and Dizio 1994; Mattar and Ostry 2007, 2010; Thoroughman and Shadmehr 2000). Our findings add to this body of work, and further argue that adaptation is local. Consistent with the behavioral evidence, previous computational studies that examine how movement errors generalize during learning have found narrow bases of representation (Donchin et al. 2003; Ingram et al. 2010; Kadiallah et al. 2012; Thoroughman and Shadmehr 2000; Thoroughman and Taylor 2005). Finally, there are also neural data that suggest localized tuning curves (Cohen and Andersen 2002; Coltz et al. 1999; Georgopoulos et al. 2007; Paz et al. 2003). These observations are consistent with the notion that neural populations encode local features of learning and should have difficulty extrapolating to completely novel circumstances. Together these findings suggest that the kind of adaptation explored in our experiments is unlikely to completely generalize to new regions of the workspace, regardless of the coordinate frame used to represent these behaviors.

Since the original study by Shadmehr and Mussa-Ivaldi (1994), a number of studies have examined how learning of dynamics generalizes across different contexts and have found contrasting evidence. Studies examining transfer from one arm to the other have shown that dynamics are transferred in an extrinsic coordinate frame when the perturbation is introduced abruptly but that there is limited transfer when the perturbation is introduced gradually such that subjects are unaware of motor errors (Criscimagna-Hemminger et al. 2003; Malfait and Ostry 2004). Another study found evidence that the coordinate frame used to represent the dynamics of hand-held tools depends on the familiarity of the tool dynamics (Ahmed et al. 2008). A recent study argues that force-field adaptation uses both extrinsic and intrinsic coordinates but generalization may be sensitive to the orientation of visual feedback (Parmar et al. 2011). There is also evidence that adaptation to a visuomotor perturbation takes place in both extrinsic and intrinsic coordinate systems (Brayanov et al. 2012). This study used a broad range of reaches to thoroughly probe the structure of generalization. Interestingly, they concluded that generalization uses a model of combined intrinsic and extrinsic coordinates in a multiplicative gain field with a local representation. Taken together, our study and this previous study provide ample evidence for multiple coordinate frames and local learning during generalization.

The suggestion that multiple coordinate frames are used is consistent with evidence in both modeling and neurophysiological studies. Recent modeling work suggests that adaptation should take place simultaneously in multiple coordinate frames (Berniker and Kording 2008, 2011) as well as multiple timescales (Kording et al. 2007; Smith et al. 2006). These results suggest considerable flexibility in the way in which dynamics may be encoded across different tasks and contexts. Neurophysiological studies of motor cortex activity during arm movement indicate that multiple features of the movements—which may be associated with different coordinate frames—are represented. Thus there is evidence for representations of hand position (Kettner et al. 1988), velocity (Georgopoulos et al. 1982; Kakei et al. 1999; Moran and Schwartz 1999), acceleration (Flament and Hore 1988), force (Evarts 1968), limb configuration (Scott and Kalaska 1995, 1997), musclelike tuning (Li et al. 2001; Sergio and Kalaska 1997), and even visual space (Georgopoulos et al. 1989).

There are noteworthy studies that have found supportive evidence for intrinsic representations during force-field generalization. In a follow-up study of intrinsic generalization (Shadmehr and Moussavi 2000), subjects were trained in a field similar to that used here and then tested for generalization in a new workspace. As in the original study by Shadmehr and Mussa-Ivaldi (1994), subjects were exposed to either a field that rotated with their limb or one that remained invariant with respect to their hand. In contrast with both experiments presented here, subjects in that experiment trained by repeatedly reaching to only four targets (roughly 144 times per target), with continuous visual feedback and without catch trials. In another similar study, subjects were highly trained, on a curl field, adapting to a single reaching direction (400 practice reaches) and tested for generalization in a single reaching direction with continuous visual feedback (Malfait et al. 2002). Two more recent studies examined generalization under essentially identical training, reaching to a single target in a curl field (Haswell et al. 2009; Orban de Xivry et al. 2011). Here again both studies found evidence for intrinsic representations. The subjects in the above experiments were highly trained relative to the subjects in our *experiments 1* and *2*, where reaches were made to multiple targets, in a direction-dependent field, with catch trials and trials without visual feedback. Furthermore, the subjects in these studies were tested for generalization in a similarly focused manner, with visual feedback, whereas our subjects were tested broadly, either without visual feedback or in error clamps. Possibly because of the focused training and testing, these previous studies found contrasting results. However, it is important to note that in the Malfait study the subjects' ability to generalize was local, and performance during reaches to a target 90° away was poor. This is consistent with our findings here.

One puzzling aspect of our results is the failure of *experiment 2* to replicate the original results of Shadmehr and Mussa-Ivaldi (1994). We attempted to match the adaptation and generalization protocols of the two experiments. Whereas our subjects demonstrated clear evidence of adaptation, not unlike the original study, we see dramatic differences in the generalization patterns between the two studies. There is the possibility that generalization is especially sensitive to minor differences in experimental protocol, the apparatus, or the robotic control algorithms that affect the force field (e.g., faster control loops, inertial compensation for robot dynamics and force feedback controllers). However, we think it is unlikely that such differences explain the difference in results. The fact that the results of our *experiments 1* and *2* are consistent in not supporting global generalization in joint-based coordinates, even though the protocol, apparatus, and control algorithms were different, suggests that our findings are robust. We also note that in *experiment 2* we ran nearly four times the number of subjects that were run in the original study. Thus our failure to replicate the original results is unlikely to be due to lack of statistical power. Given these considerations, we propose that the findings presented here are an accurate depiction of how human subjects generalize.

The paper by Shadmehr and Mussa-Ivaldi (1994) has been exceptionally important for the development of the motor control field (with over 1,200 citations at the time of this writing). It established the use of robots to probe adaptation and learning; it showed that humans adapt by predicting perturbations; it formalized the search for the coordinate system of these internal models; and it concluded that generalization takes place using intrinsic coordinates. We only take issue with this last finding. Although we present evidence for multiple coordinate frames being used simultaneously or for a decaying generalization pattern, it seems fair to suggest that the coordinate system of generalization and internal models may be far more complicated than currently assumed.

## GRANTS

This work was supported by the Wellcome Trust, the Biotechnology and Biological Sciences Research Council (BBSRC), the Human Frontier Science Program (HFSP), the Canadian Institutes of Health Research, and National Institute of Neurological Disorders and Stroke (NINDS) Grant 1R01 NS-063399. *Experiment 2* was performed in the Robotics Laboratory of the Rehabilitation Institute of Chicago with the support of NINDS Grant 2R01 NS-035673 to F. A. Mussa-Ivaldi.

## DISCLOSURES

No conflicts of interest, financial or otherwise, are declared by the author(s).

## AUTHOR CONTRIBUTIONS

Author contributions: M.B., D.W.F., J.R.F., D.M.W., and K.K. conception and design of research; M.B. and D.W.F. performed experiments; M.B. and D.W.F. analyzed data; M.B., D.W.F., J.R.F., D.M.W., and K.K. interpreted results of experiments; M.B., D.W.F., and D.M.W. prepared figures; M.B., D.W.F., J.R.F., D.M.W., and K.K. drafted manuscript; M.B., D.W.F., J.R.F., D.M.W., and K.K. edited and revised manuscript; M.B., D.W.F., D.M.W., and K.K. approved final version of manuscript.
